# TRIM38 Negatively Regulates TLR3-Mediated IFN-β Signaling by Targeting TRIF for Degradation

**DOI:** 10.1371/journal.pone.0046825

**Published:** 2012-10-08

**Authors:** Qinghua Xue, Zhuo Zhou, Xiaobo Lei, Xinlei Liu, Bin He, Jianwei Wang, Tao Hung

**Affiliations:** 1 MOH Key Laboratory of Systems Biology of Pathogens, Institute of Pathogen Biology, Chinese Academy of Medical Sciences and Peking Union Medical College, Beijing, People’s Republic of China; 2 Department of Microbiology and Immunology, College of Medicine, University of Illinois, Chicago, Illinois, United States of America; University of Tennessee Health Science Center, United States of America

## Abstract

Toll-like receptors (TLRs) mediated immune response is crucial for combating pathogens and must be tightly controlled. Tripartite motif (TRIM) proteins are a family of proteins that is involved in a variety of biological and physiological processes. Some members of the TRIM family are important in the regulation of innate immunity. Although it has been shown that TRIM38 negatively regulates innate immunity, the mechanisms by which it does so have not been fully addressed. In this study, we demonstrated that TRIM38 negatively regulates Toll-like receptor 3 (TLR3)-mediated type I interferon signaling by targeting TIR domain-containing adaptor inducing IFN-β (TRIF). We found that overexpression of TRIM38 inhibits TLR3-mediated type I interferon signaling, whereas knockdown of TRIM38 has the reverse effects. We further showed that TRIM38 targets TRIF, a critical adaptor protein downstream of TLR3. TRIF is co-immunoprecipitated with TRIM38, and domain mapping experiments show that PRYSPRY of TRIM38 interacts with the N-terminus of TRIF. Overexpression of TRIM38 decreased expression of overexpressed and endogenous TRIF. This effect could be inhibited by MG132 treatment. Furthermore, the RING/B-box domain of TRIM38 is critical for K48-linked polyubiquitination and proteasomal degradation of TRIF. Collectively, our results suggest that TRIM38 may act as a novel negative regulator for TLR3-mediated type I interferon signaling by targeting TRIF for degradation.

## Introduction

The innate immune response against invading pathogens relies on sensing of pathogen-associated molecular patterns (PAMPs) by germline-encoded pattern-recognition receptors (PRRs) [1], [2]. These PRRs include Toll-like receptors (TLRs), RIG-I-like receptors (RLRs), NOD-like receptors (NLRs), and C-type lectin receptors (CLRs) [2], [3], [4], [5]. Nucleic acids of pathogens are the major ligands of these receptors. Viral double-stranded RNA (dsRNA) is recognized by endosome TLR3 or by cytoplasmic sensors such as retinoic acid inducible gene I (RIG-I) and melanoma differentiation-associated gene 5 (MDA5) [6], [7]. Each PRR recruits downstream adaptor protein that determines the type of response by activating distinct transcription factors, which eventually induce type I interferons (IFNs) and cytokines [8], [9]. Mitochondrial antiviral signaling protein (MAVS) interacts with RIG-I/MDA5 and mediates innate immune response by inducing the production of type I IFNs [4], [10]. Toll/interleulin-1 receptor (TIR) domain-containing adaptor inducing IFN-β (TRIF) is the single adaptor molecule of TLR3. TRIF mediates activation of transcription factor IFN regulatory factor (IRF3), nuclear factor-kappa B (NF-κB) and activator protein 1 (AP-1), leading to the induction of IFNs, cytokines, and maturation of dendritic cells (DCs) [11], [12], [13]. Upon stimulation of TLR3, TRIF transiently couples with TLR3 and then dissociates from the receptor to recruit the downstream kinase complex via TNF receptor-associated factor 3 (TRAF3), leading to the production of type I IFNs [11], [14]. Additionally, TRIF recruits receptor-interacting protein 1 (RIP1) and TRAF6 to induce NF-κB activation [9], [15].

**Figure 1:**
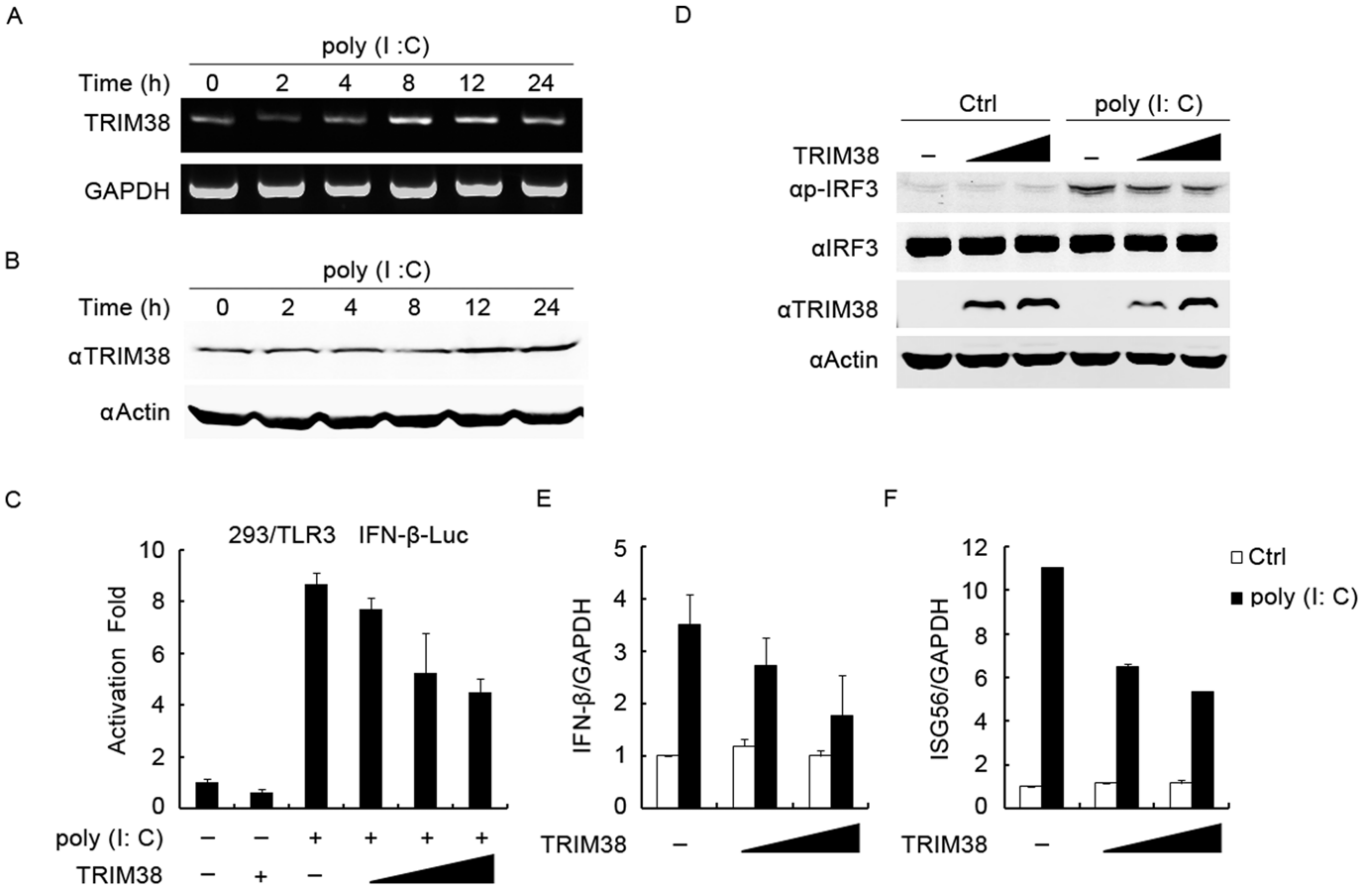
Effects of TRIM38 on TLR3-induced IFN-β signaling. (A) Expression of TRIM38 mRNA in HeLa cells treated with 100 µg/ml poly(I:C). At indicated time points, cells were harvested, and total RNA was prepared and analyzed by RT-PCR. GAPDH mRNA expression was assessed as an internal control. (B) Expression of TRIM38 protein in HeLa cells treated with 100 µg/ml poly(I:C). At indicated time points, cells were harvested and analyzed by immunoblot using the indicated antibodies. (C) Effect of TRIM38 overexpression on poly(I:C)-induced activation of IFN-β. 293T/TLR3 cells were transfected with an IFN-β-luc plasmid and TRIM38 plasmid (0, 50, 200, and 500 ng). Twenty-four hours after transfection, cells were incubated with 100 µg/ml of poly(I:C) for 4 h, and then luciferase assays were performed. (D) Effect of TRIM38 overexpression on IRF3 phosphorylation. HeLa cells were transfected with TRIM38 plasmid (0, 0.5 and 2 µg). Twenty-four hours after transfection, cells were left untreated or incubated with 100 µg/ml poly(I:C) for 4 h. Immunoblot analysis was performed using the indicated antibodies. (E, F) Effect of TRIM38 overexpression on poly(I:C)-induced transcription of IFN-β and ISG56. HeLa cells were transfected with TRIM38 plasmid (0, 0.5, and 2 µg). Twenty-four hours after transfection, cells were left untreated or incubated with 100 µg/ml of poly(I:C) for 4 h, then total RNA was extracted and quantitative real-time PCR were performed to analyze gene expression.

The innate immune response is essential for controlling microbial infection. However, over-activation of the innate immune response may lead to chronic inflammation and autoimmune diseases [16], [17]. Therefore, signal transduction in the immune response must be under tight regulation to maintain immune balance. Multiple mechanisms that limit the immune response have been identified, such as degradation, phosphorylation or sequestration of signaling molecules [18]. Many negative regulators reportedly exert their functions by targeting signaling molecules for ubiquitin-mediated proteasomal degradation [19], [20]. For example, RING-finger protein 5 (RNF5), an E3 ubiquitin ligase, promotes degradation of STING (stimulator of interferon genes, also named MITA) [21], [22]; Cbl-b negatively regulates TLR signaling through mediating degradation of myeloid differentiation primary response gene 88 (MyD88) and TRIF [23].

The tripartite motif-containing (TRIM) proteins are characterized by the tripartite motif. The TRIM motif comprises a RING domain, a B-box domain and a coiled-coil region [24]. More than 70 members of the TRIM family, which may be involved in a variety of biological and physiological processes, have been identified in humans. As the RING domain can mediate ubiquitin conjugation, many TRIM proteins have been identified as E3 ubiquitin ligases [24], [25]. Particularly, TRIM5α, TRIM21, and TRIM22 act as E3 ubiquitin ligases to affect viral replication directly [26], [27], [28]. In addition, PRYSPRY domain, a typical C-terminal domain of TRIM, mediates protein-protein interaction and plays a critical role in TRIM functions [29]. Recent research suggests that some TRIM proteins are involved in innate immune response [30]. However, it is not clear if additional TRIMs regulate this process.

**Figure 2:**
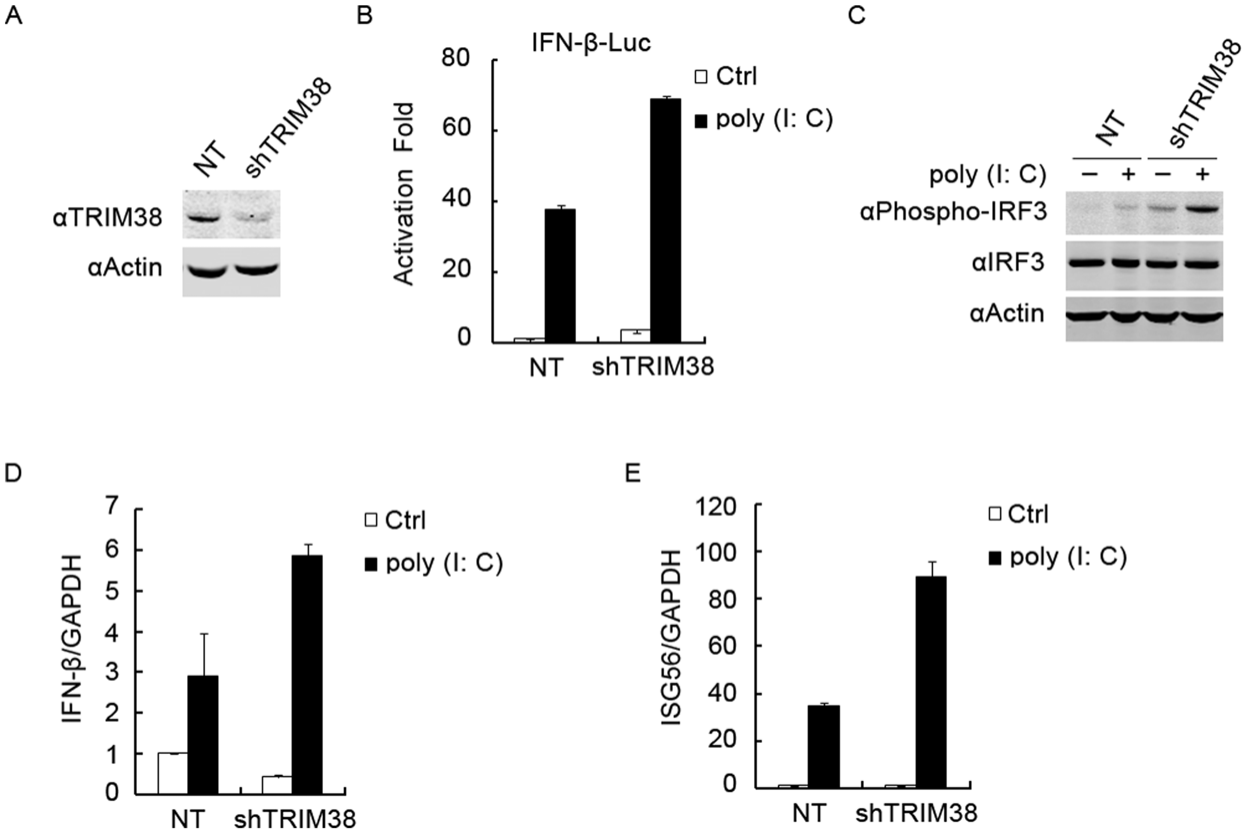
Effects of TRIM38 knockdown on TLR3-induced IFN-β activation. (A) TRIM38 protein level in 293/TLR3 cell line stably expressing TRIM38-specific or non-targeting shRNA. 293/TLR3 cells were transfected with TRIM38-specific shRNA or non-targeting shRNA (NT) followed by puromycin selection and cloning. Screened cell populations were analyzed by immunoblot with an antibody specific for TRIM38. (B) Effects of TRIM38 knockdown on poly(I:C)-induced IFN-β activation. TRIM38 knockdown or control 293/TLR3 cells were transfected with IFN-β-luc plasmid. Twenty-four hours after transfection, cells were left untreated or stimulated with 100 µg/ml of poly(I:C) for 4 h before luciferase analysis was performed. (C) Effects of TRIM38 knockdown on poly(I:C)-induced IRF3 phosphorylation. TRIM38 knockdown or control 293/TLR3 cells were incubated with 100 µg/ml of poly(I:C) for 4 h before the immunoblot analysis was performed. (D, E) Effects of TRIM38 knockdown on poly(I:C)-induced transcription of IFN-β and ISG56 genes. Indicated cells were stimulated with poly(I:C) for 4 h, then total RNA was extracted for real-time PCR analysis. Similar results were obtained from three independent experiments.

TRIM38 is reported to negatively regulate innate immunity [31]. However, the molecular mechanisms by which TRIM38 negatively regulate innate immunity are not fully understood. In this report, we demonstrate that TRIM38 negatively regulates TLR3-mediated type I interferon signaling by targeting TRIF for degradation. Overexpression or knockdown of TRIM38 could inhibit or promote TLR3-mediated type I interferon signaling respectively. Mechanistically, TRIM38 is shown to interact with TRIF and mediate TRIF degradation via the ubiquitin-proteasome pathway. Further, we suggest that the RING/B-box domain of TRIM38 is critical for K48-linked polyubiquitination and proteasomal degradation of TRIF. Our findings may provide new insights in the mechanisms for anti-viral innate immune responses that are fine tuned by TRIM family proteins.

**Figure 3:**
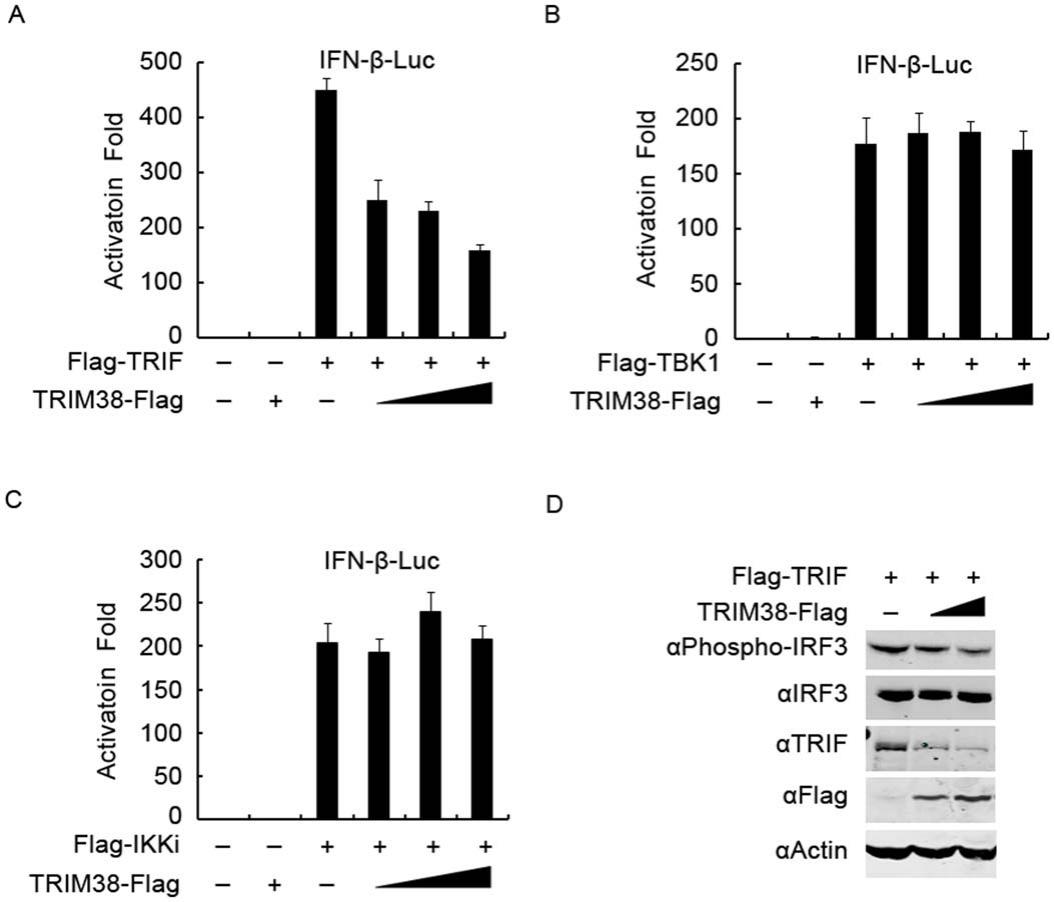
TRIM38 targets TRIF. (A–C) Effects of TRIM38 on TRIF, TBK1, or IKKi-induced IFN-β activation. 293T cells were transfected with an IFN-β-luc plasmid, together with a plasmid expressing TRIF (A), TBK1 (B), or IKKi (C), and a TRIM38 plasmid (0, 50, 100, and 200 ng), respectively. Luciferase assays were performed after 24 h post-transfection. (D) Effect of TRIM38 on TRIF-mediated IRF3 phosphorylation. 293T cells were transfected with a TRIF plasmid and a TRIM38 plasmid (0, 0.5, and 2 µg). Twenty-four hours post transfection, cell lysates were analyzed by immunoblot with indicated antibodies.

## Materials and Methods

### Cell Culture and Reagents

293T (ATCC, CRL-11268) cells, and HeLa (ATCC, CCL-2) cells were cultured in Dulbecco’s modified Eagle’s medium (DMEM, HyClone, Logan, UT) supplemented with 10% fetal bovine serum (FBS, HyClone) and antibiotics. 293 cells stably expressing TLR3 (293/TLR3) were a gift form Dr. Zhengfan Jiang (Peking University, Beijing, China) [32].

MG132, NH_4_Cl, Z-VAD-FMK, polyinosinic: polycytidylic acid [poly(I:C)] were purchased from Sigma (St. Louis, MO).

Antibodies against Flag, Myc, HA, and β-actin were purchased from Sigma; rabbit anti-TRIF and anti-TRAF3 antibodies were from Cell Signaling Technology (Danvers, MA). Goat anti-TRIF antibody was from R&D Systems (Minneapolis, MN). Rabbit antibodies against IRF3 and phospho-IRF3 (pS386) were from Epitomics (Burlingame, CA). Rabbit anti-TRIM38 antibody was from Sigma. Rabbit K48-linkage ubiquitin antibody was from Millipore (Billerica, MA). The IRDye 800-labeled IgG or IRDye 680-labeled IgG secondary antibodies were from LI-COR Biosciences (Lincoln, NE).

**Figure 4:**
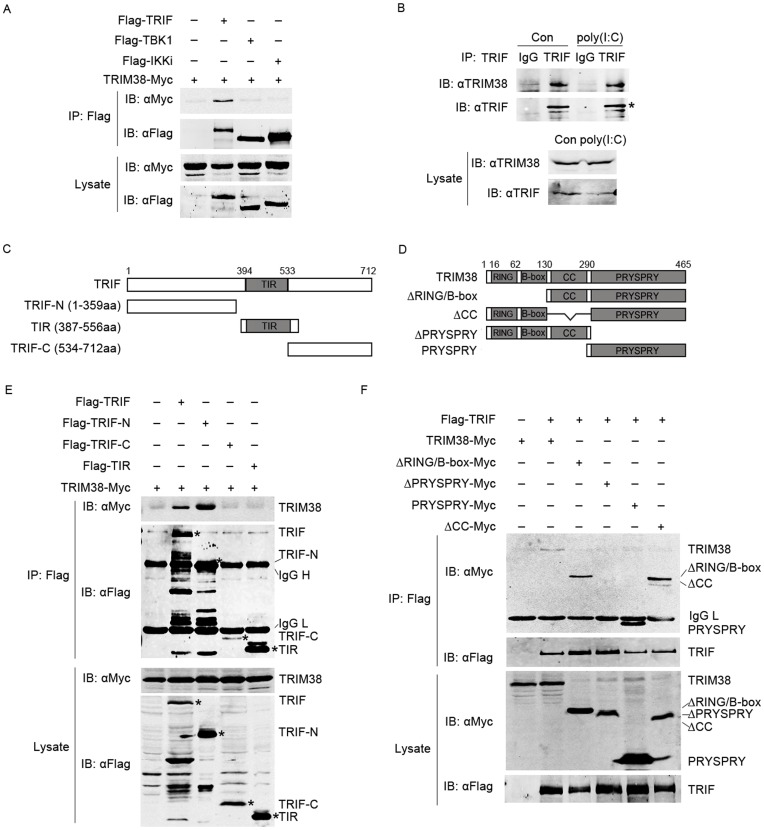
Interaction of TRIM38 and TRIF. (A) TRIM38 specifically interacts with TRIF. 293T cells transfected with plasmids expressing TRIM38-Myc and Flag-TRIF, Flag-TBK1, or Flag-IKKi. After 24 h, cell lysates were immunoprecipitated using anti-Flag agarose beads. The lysates and immunoprecipitates were analyzed by immunoblot with anti-Myc and anti-Flag antibodies. (B) Endogenous interaction of TRIF with TRIM38. Hela cells were left untreated or treated with poly(I:C) for 4 h. Cell lysates were immunoprecipitated with goat anti-TRIF antibody or control goat IgG and analyzed with rabbit anti-TRIM38 or rabbit anti-TRIF antibodies. (C, D) Schematic presentations of truncation mutants of TRIF (C) and TRIM38 (D). (E) TRIF interacts with TRIM38 through its N-terminus. 293T cells were transfected with Myc-TRIM38 together with full length Flag-tagged TRIF or TRIF truncation mutants. The immunoprecipitations were performed using anti-Flag agrose beads. Immunoblot analysis was carried out similarly as in (A). (F) TRIM38 is associated with TRIF through its PRYSPRY domain. Experiments were performed as described in panel (E). The co-immunopricipitated proteins were marked by asterisks.

### Plasmid Construction

The plasmids expressing Flag-tagged proteins including Flag-TRIF, Flag-TBK1, and Flag-IKKi have been described elsewhere [33], [34]. The plasmid expressing full length TRIM38 was purchased from Origene (Rockville, MD). The TRIF mutants, including TRIF-N (aa 1–359), TRIF-C (aa 534–712), TIR (aa 387–556), and TRIF (D281ED289E), as well as the TRIM38 mutants, including ΔRING/B-box (aa 130–465), ΔPRYSPRY (aa 1–274), ΔCC (deleted aa 130–290) and PRYSPRY (aa 274–465) were generated using a Site-Directed Mutagenesis Kit (Stratagene, La Jolla, CA).

**Figure 5:**
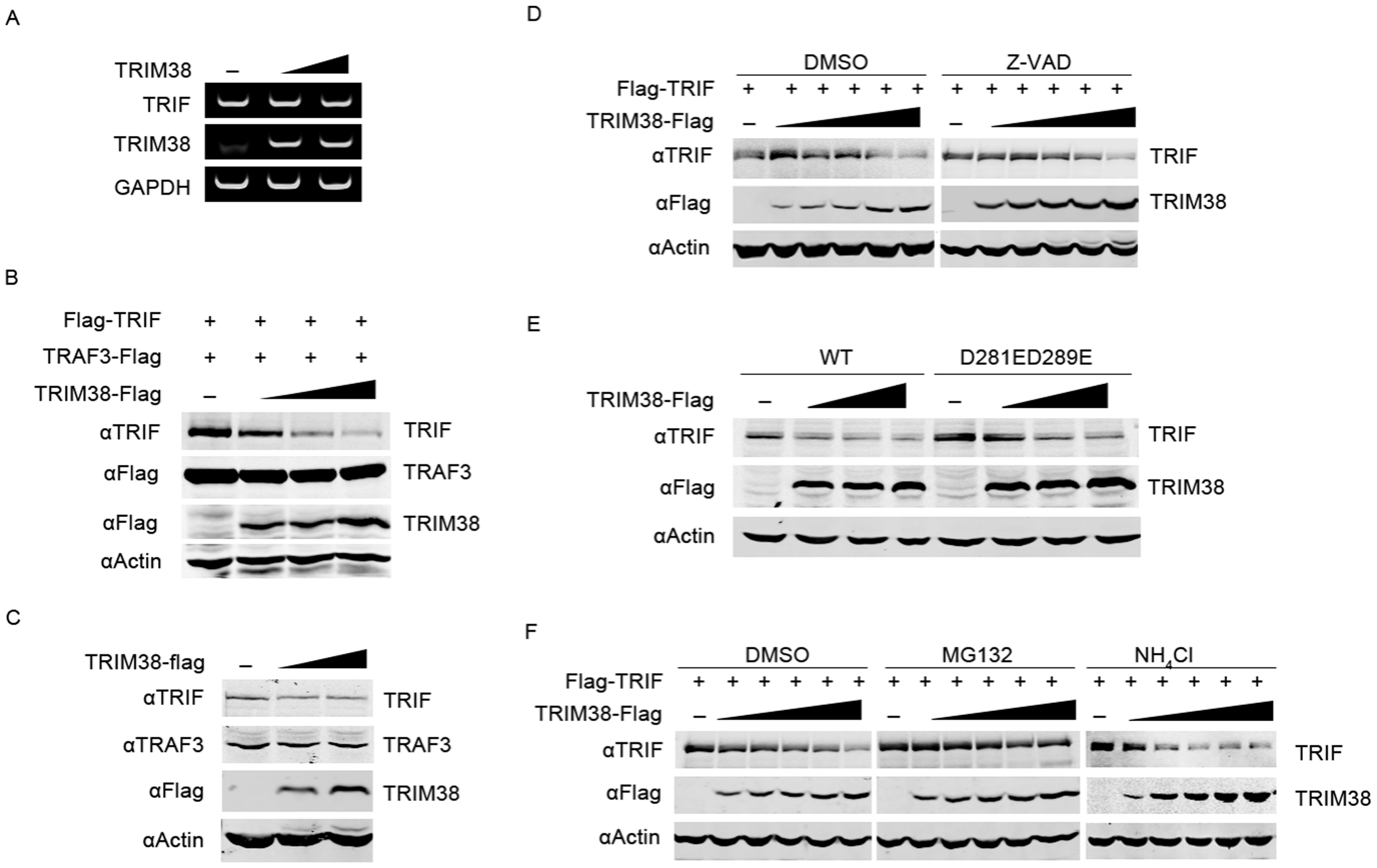
TRIM38 induces TRIF degradation through proteasomal pathway. (A) Overexpression of TRIM38 does not affect endogenous TRIF mRNA expression. HeLa cells were transfected with increasing amounts of a TRIM38-Flag plasmid (0, 0.5, and 2 µg). After 48 h, total RNA was prepared and used for RT-PCR analysis of the indicated genes. GAPDH was used as an internal control. (B) Overexpression of TRIM38 reduces TRIF protein. 293T cells were transfected with plasmids expressing Flag-TRIF or TRAF3-Flag, together with increasing amounts of TRIM38-Flag plasmid (0, 50, 100, and 200 ng). After 24 h, immunoblot analysis was performed with indicated antibodies. (C) Overexpression of TRIM38 reduces endogenous TRIF. HeLa cells were transfected with increasing amounts of TRIM38-Flag plasmid (0, 0.5, and 2 µg). Fourty-eight hours after transfection, immunoblot analysis was performed using indicated antibodies. (D) Caspase inhibitor does not inhibit TRIM38-mediated TRIF degradation. 293T cells were transfected with Flag-TRIF plasmid and increasing amounts of TRIM38-Flag plasmid (0, 10, 50, 100, 150, and 200 ng) for 6 h. Then cells were treated with DMSO (negative control) or 5 µM Z-VAD-FMK for 16 h before immunoblot analysis was performed. (E) Overexpression of TRIM38 promotes degradation of caspase-resistant TRIF. 293T cells were transfected with plasmids expressing Flag-TRIF or TRIF mutant carrying D284E and D289E substitutions, together with increasing amounts of TRIM38-Flag plasmid (0, 50, 100, and 200 ng). After 24 h, immunoblot analysis was performed. (F) TRIM38 promotes proteasomal degradation of TRIF. 293T cells were transfected with a Flag-TRIF plasmid and increasing amounts of TRIM38-Flag plasmid (0, 10, 50, 100, 150, and 200 ng) for 6 h. Then cells were treated with DMSO, 0.1 µM MG132, or 10 mM NH_4_Cl for 16 h before immunoblot analysis was performed.

### Generation of TRIM38 Knockdown Cell Line

293/TLR3 cells were transfected with non-targeting (NT) or TRIM38-specific shRNA. After selected with puromycin (1 µg/ml), single cells were isolated using limit dilution and screened for maximal knockdown of TRIM38 using immunoblot analyses. TRIM38-specific shRNAs were from Origene. The sequence of TRIM38-specific shRNA is 5′-ATCGGAGACAAGTGACTCGTGGATACACC-3′, and the sequence of the non-targeting shRNA is 5′-GCACTACCAGAGCTAACTCAGATAGTACT-3′.

**Figure 6:**
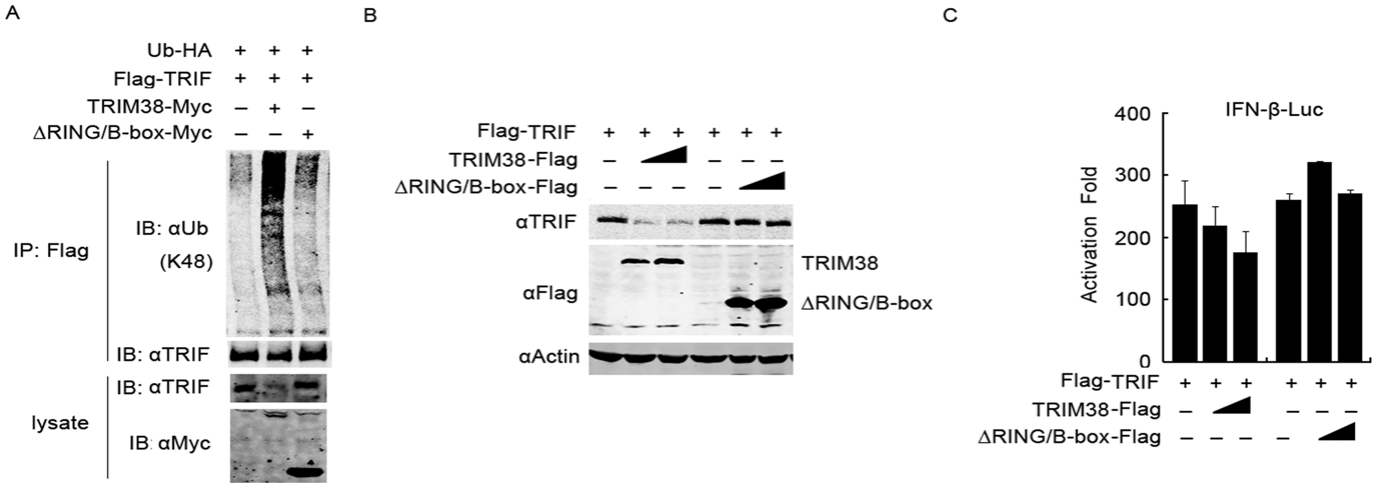
RING/B-box of TRIM38 is critical for TRIF degradation. (A) TRIM38 catalyzes K48-linked ubiquitination of TRIF. 293T cells were transfected with plasmids expressing Myc-tagged full-length or RING/B-box domain deleted (ΔRING/B-box) TRIM38, Flag-TRIF, and HA-ubiquitin plasmids. At 24 h post-transfection, cell lysates were denatured and immunoprecipitated using anti-Flag agrose beads. Immunoblot analysis was performed using an antibody specific against K48-linkage polyubiquitin. (B) Effect of TRIM38ΔRING/B-box mutant on TRIF degradation. 293T cells were transfected with Flag-TRIF plasmid and Flag-tagged full length TRIM38 or TRIM38ΔRING/B-box mutant plasmid (0, 50, and 100 ng). Twenty-four hours after transfection, immunoblot analysis using the indicated antibodies was performed. (C) Effect of TRIM38ΔRING/B-box mutant on TRIF-induced IFN-β promoter activation. 293T cells were transfected with IFN-β-Luc plasmid, Flag-TRIF plasmid, together with increasing amounts plasmid expressing of Flag-tagged full length TRIM38 or TRIM38ΔRING/B-box mutant (0, 50, and 100 ng). Luciferase assays were performed 24 h after transfection.

### Reporter Assays

293T or 293/TLR3 cells cultured in 24-well plates (2.5×10^5^ cells per well) were transfected with a control plasmid or plasmids expressing Flag-TRIF, Flag-TBK1, Flag-IKKi, and TRIM38 plasmids, along with pGL3-IFN-β-luc, and pRL-SV40 [34] using Lipofectamine 2000 (Invitrogen, Carlsbad, CA). At 24 h post-transfection, cells were lysed, and luciferase activities were determined using a dual luciferase reporter gene assay kit (Promega, Madison, WI). The firefly luciferase activities were normalized to the Renilla luciferase activities.

### RT-PCR and Real-time PCR

Total RNA was extracted using TRIzol reagent (Invitrogen) and treated with DNase I (Pierce, Rockford, IL). Extracted RNA was used as template for reverse transcription using the Superscript cDNA synthesis kit (Invitrogen), according to the manufacturer’s instructions. These cDNA samples were then subjected to PCR analysis using primers specific for detection of IFN-β and ISG56 [34]. Quantitative real-time PCR was performed using SYBR green kit (Takara Bio, Otsu, Japan), according to the manufacturer’s instructions. Expression of IFN-β and ISG56 mRNA was normalized to GAPDH mRNA expression.

### Immunoprecipitation

293T cells were seeded in 10 cm^2^ dishes (8×10^6^ per dish). The next day, cells were transfected with indicated plasmids using Lipofectamine 2000 (Invitrogen). Twenty-four hours post-transfection, cells were harvested and lysed with RIPA buffer containing 25 mM Tris-HCl (pH7.4), 150 mM NaCl, 1 mM EDTA, 1% NP40, 0.25% sodium deoxycholate and protease inhibitor cocktail (Roche, Indianapolis, IN). For immunoprecipitation assays, cell lysates were incubated with anti-Flag M2-agrose beads (Sigma) overnight at 4°C with gentle shaking. The beads were washed four times with lysis buffer containing 0.2% NP40. The immunoprecipitates were boiled in 2×SDS loading buffer. For immunoprecipitation of endogenous TRIF, Hela cells were treated with 100 µg/ml poly(I:C) for 4 hours before cells were harvested and lysed in RIPA buffer. Cell lysates were incubated with 10 µg goat anti-TRIF antibody or control IgG overnight at 4°C. Then, 30 µl prewashed protein A/G beads were added to each sample and incubated at 4°C. After 4 h, the beads were washed with cold PBS four times, and were boiled in 2×SDS loading buffer. To detect ubiquitination of TRIF, 10 mM *N*-ethylmaleimide (Sigma) and 1% SDS were included in the lysis buffer. The cell lysates were denatured at 90°C for 5 min. Then the lysates were diluted with RIPA buffer until the concentration of SDS was decreased to 0.1%. Then immunoprecipitation assays were performed, and the samples were analyzed by immunoblot with indicated antibodies.

## Results

### Overexpression of TRIM38 Inhibits TLR3-mediated IFN-β Signaling

Recently, TRIM38 was shown to negatively regulate TLR-mediated NF-κB activation [31]. However, its role in the TLR-medaited pathway has not been fully elucidated. To determine if TRIM38 is involved in the TLR3-medaited pathway, we measured the mRNA and protein expression of TRIM38 in HeLa cells exposed to poly(I:C), a synthetic TLR3 agonist. Expression of TRIM38 mRNA and protein were induced at different time points after exposure of cells to poly(I:C) (Fig. 1A and B), suggesting that TRIM38 may be involved in TLR3-mediated signaling. We next investigated the effects of TRIM38 overexpression on TLR3-mediated activation of IFN-β. 293T/TLR3 cells, which stably express TLR3 [32], were transfected with an IFN-β promoter-driven luciferase reporter (IFN-β-luc) plasmid and different amounts of a TRIM38 plasmid. After 24 h, cells were incubated with poly(I:C) for 4 h, and then luciferase activities were measured. In parallel, expression of TRIM38 was verified by immunoblot analyses. Overexpression of TRIM38 inhibited poly(I:C)-induced activation of the IFN-β promoter in a dose-dependent manner (Fig. 1C and Fig. S1). We also assessed the phosphorylation of IRF3, a hallmark of IRF3 activation that is required for IFN-β signaling. Overexpression of TRIM38 inhibited poly(I:C)-induced phosphorylation of IRF3 in a dose-dependent manner in HeLa cells (Fig. 1D). Moreover, we examined if overexpression of TRIM38 alters poly(I:C)-induced mRNA expression of IFN-β and IFN-stimulated genes (ISGs). Consistently, quantitative real-time PCR analyses showed that overexpression of TRIM38 attenuated poly(I:C)-induced mRNA expression of IFN-β and ISG56 genes (Fig. 1E and F). Taken together, these findings suggest that TRIM38 negatively regulates TLR3-mediated IFN-β signaling.

### Knockdown of TRIM38 Potentiates TLR3-mediated IFN-β Activation

To confirm the role of TRIM38 in TLR3-mediated signaling, we investigated whether knockdown of endogenous TRIM38 affects TLR3-mediated IFN-β activation. To achieve constant knockdown of TRIM38, we generated the 293/TLR3 cell line that stably expresses TRIM38-specific or non-targeting shRNA. Cells transfected with the TRIM38-specific shRNA plasmid showed ∼70% reduction of TRIM38 expression than control cells (Fig. 2A). We used reporter assays to determine IFN-β activation in these cell lines. Knockdown of TRIM38 potentiated poly(I:C)-induced activation of IFN-β (Fig. 2B). We then analyzed the activation of IRF3 in both TRIM38 knockdown and control cells. Immunoblot analysis showed that poly(I:C)-induced IRF3 phosphorylation was significantly higher in TRIM38 knockdown cells than that in control cells (Fig. 2C), indicating that TRIM38 inhibits TLR3-mediated IRF3 activation. Furthermore, we used quantitative real-time PCR to assess expression of IFN-β and ISG56 genes in the TRIM38 knockdown cells. Exposure to poly(I:C) led to a 2–3 fold increase in IFN-β and ISG56 mRNA expression 4 h post-infection in the TRIM38 knockdown cells compared to control cells (Fig. 2D and E). Collectively, these results show that TRIM38 negatively regulates TLR3 signaling.

### TRIM38 Targets TRIF

To investigate the potential target of TRIM38 in TLR3-mediated IFN-β signaling, we first determined the inhibitory effect of TRIM38 on IFN-β activation induced by various signaling molecules in TLR3 pathway. We transfected 293T cells with plasmids encoding TRIF, TBK1, or IKKi, together with increasing amounts of TRIM38 plasmid and an IFN-β-luc plasmid. After 24 h, IFN-β promoter activity was determined using luciferase assay, and the protein expression was analyzed by immunoblot assays (Fig. S2). Overexpression of TRIM38 inhibited TRIF-induced IFN-β activation in a dose-dependent manner, but did not affect TBK1 or IKKi-induced IFN-β activation (Fig. 3A, B, and C). Furthermore, we determined whether TRIM38 inhibits TRIF-induced IRF3 phosphorylation. Immunoblot analysis shows that TRIM38 inhibited TRIF-triggered IRF3 phosphorylation in a dose-dependent manner (Fig. 3D), indicating that TRIF might be a target for TRIM38.

### TRIM38 Interacts with TRIF

To further investigate the underlying mechanism of TRIM38 in poly(I:C)-induced IFN-β activation, we examined the interaction between TRIM38 and TRIF, TBK1 or IKKi. Results of immunoprecipitation experiments indicate that TRIM38 interacts with TRIF, but not with TBK1 or IKKi (Fig. 4A). This finding was further confirmed by endogenous co-immunoprecipitation experiments (Fig. 4B).

We then mapped the region that is responsible for TRIM38-TRIF association. Various truncatants of TRIM38 and TRIF were generated (Fig. 4C and D), and the interactions were analyzed using immunoprecipitation. The N-terminus of TRIF interacts with TRIM38, whereas TIR region and the C-terminus of TRIF do not interact with TRIM38 (Fig. 4E). Moreover, TRIF interacts with the PRYSPRY domain of TRIM38, and deletion of PRYSPRY domain disrupts TRIF-TRIM38 interaction (Fig. 4F). The results suggested that N-terminus of TRIF and PRYSPRY domain of TRIM38 are the critical regions that mediate TRIM38-TRIF interaction (Fig. 4F).

### TRIM38 Promotes TRIF Degradation through Proteasome Pathway

Notably, the protein levels of TRIF decreased when TRIF was co-expressed with TRIM38 (Fig. 3D). Based on this observation, we speculated that TRIM38 might inhibit TRIF gene expression and/or promote TRIF degradation. To test this, we first examined whether TRIF mRNA expression was affected by TRIM38 overexpression. HeLa cells were transfected with increasing amounts of TRIM38 plasmid, with approximately 70% transfection efficiency (data not shown). At 48 h post transfection, total RNA was extracted and RT-PCR was performed. The amount of TRIF mRNA remained constant when TRIM38 was increasingly overexpressed (Fig. 5A).This indicates that TRIM38 may not downregulate TRIF at the transcription level.

We next investigated the effect of TRIM38 overexpression on TRIF protein level. HeLa cells were transfected with control or TRIF plasmid, together with increasing amounts of TRIM38 plasmid. At 48 h post transfection, cells were lysed and the expression of TRIF protein was examined using immunoblot assays. The protein levels of overexpressed TRIF (Fig. 5B) and endogenous TRIF (Fig. 5C) decreased in the presence of overexpressed TRIM38. In contrast, overexpression of TRIM38 did not affect the level of TRAF3, another critical signaling protein downstream of TRIF. Furthermore, we found that the protein level of endogenous TRIF decreased slightly upon poly(I:C) stimulation at various time points (Fig. S3). Taken together, these findings suggest that TRIM38 could specifically target TRIF for protein degradation.

Cellular caspases can cleave TRIF [35]. To test whether TRIM38 mediates TRIF degradation through caspase cleavage, we examined if the caspase inhibitor Z-VAD-FMK blocks TRIM38-mediated degradation of TRIF. The decrease of TRIF protein was not inhibited by Z-VAD-FMK (Fig. 5D), indicating that caspases may be not involved in this process. To confirm this, we analyzed a TRIF mutant carrying D281E and D289E substitutions, which is resistant to caspase cleavage [35]. Similar to wild-type TRIF, cleavage-resistant TRIF decreased in a dose-dependent manner upon TRIM38 overexpression (Fig. 5E), indicating that caspases do not contribute to TRIM38-mediated degradation of TRIF. Next, we used specific inhibitors of the proteasome or lysosome pathway to determine which degradation machinery could be associated with TRIM38-mediated degradation of TRIF. We found that the proteasome inhibitor MG132, but not the lysosome inhibitor NH_4_Cl, blocks TRIM38-mediated degradation of TRIF (Fig. 5F), indicating that TRIM38 mediates TRIF degradation through the proteasome pathway rather than the lysosome pathway.

### TRIM38 Promotes K48-linked Polyubiquitination and Proteasomal Degradation of TRIF

Considering that TRIM38 may act as an E3 ubiquitin ligase [36] and that K48-linked ubiquitin chains target proteins for degradation by the proteasome pathway [37], we examined if TRIM38 could promote K48-linked polyubiquitination of TRIF. We transfected 293T cells with control or TRIM38-Myc plasmid, together with HA-Ub and Flag-TRIF plasmids. We then immunoprecipitated TRIF using anti-Flag antibody and examined the conjugation of ubiquitin using an antibody specific for K48-linked polyubiquitin. The K48-linked polyubiquitination of TRIF was significantly induced by TRIM38 overexpression (Fig. 6A). In contrast, when TRIF was co-expressed with a TRIM38 mutant lacking the RING/B-box domain, a critical region for catalyzing ubiquitination, TRIF was not significantly ubiquitinated (Fig. 6A). These results indicate that TRIM38 might mediate K48-linked polyubiquitination of TRIF through the RING/B-box domain. Furthermore, we tested the effects of RING/B-box-deleted TRIM38 on TRIF degradation and TRIF-induced activation of IFN-β. The RING/B-box deletion mutant of TRIM38 did not mediate TRIF degradation or inhibit TRIF-induced activation of IFN-β (Fig. 6B and C), suggesting that RING/B-box domain is critical for TRIF degradation mediated by TRIM38. Together, these results indicate that TRIM38 promotes K48-linked polyubiquitination and proteasomal degradation of TRIF protein.

## Discussion

Type I IFNs play a critical role in limiting the spread of viral infection [1], [17], [38]. However, the production of type I IFNs must be tightly regulated to maintain immune balance. Here, we identified TRIM38 as a negative regulator of TLR3-mediated production of type I IFNs. Furthermore, our findings suggest that TRIM38 targets TRIF and promotes degradation of TRIF through K48-linked polyubiquitination. Therefore, we postulate that TRIM38 limits the excessive production of type I IFNs in response to viral infection by mediating degradation of TRIF.

Ubiquitination plays an essential role in the regulation of innate immunity. The TRIM family is one of the largest families of RING-containing E3 ubiquitin ligases, and growing evidence suggests that many TRIM proteins play an important role in the regulation of innate immunity [30]. For example, TRIM25 promotes K63-linked polyubiquitination of RIG-I and triggers antiviral signaling [39]. TRIM21 negatively regulates production of type I IFN-β by mediating proteasomal degradation of IRF3 and IRF7 [40], [41]. TRIM27 (RFP) negatively regulates antiviral and inflammatory responses by targeting IKKs [42]. TRIM56 facilitates dsDNA-triggered signaling by targeting STING for K63-linked polyubiquitination [43]. More recently, it was reported that TRIM38 negatively regulates TLR-induced activition of NF-κB. That report suggests that TRIM38 targets TRAF6 and promotes K48-linked ubiquitination of TRAF6 for degradation, thus limiting the production of pro-inflammatory cytokines [31]. Here, we show that TRIM38 targets TRIF for degradation, implying a novel mechanism by which TRIM38 negatively regulates the innate immune response mediated by TLR3.

TRIF is the critical adaptor of the TLR3-mediated immune response, coupling TLR3 and downstream signaling molecules to trigger production of type I IFNs [12], [13], [44]. As TRIF plays a central role in TLR3-mediated signaling, it has been suggested that TRIF is a regulatory target for both the virus and host. On the one hand, viruses target TRIF for degradation to support their sufficient replication. For example, hepatitis C virus protease NS3-4A [45] and coxsackievirus B/enterovirus 71 3C^pro^ protease target TRIF for cleavage [33], [46]. On the other hand, TRIF is tightly regulated by host factors to prevent excessive immune response. For example, SARM negatively regulates TRIF-dependent TLRs signaling [47], and Integrin CD11b negatively regulates TLR-induced inflammatory responses by targeting MyD88 and TRIF for degradation [23]. Here, we showed that TRIM38 interacts with TRIF through the PRYSPRY domain, and promotes K48-linked polyubiquitination and proteasomal degradation of TRIF through the RING/B-box domain. We speculate a model that upon stimulation of TLR3, the protein level of TRIM38 is induced, which subsequently associates with and mediates the degradation of TRIF, thus fine-tuning both inflammation and innate immune response to pathogens.

Interestingly, it seems that PRYSPRY domain play an important role in innate immune regulation. The rhesus monkey TRIM5α blocks HIV-1 infection by recognizing HIV-1 core via PRYSPRY domain [48], and the SPRY domain of TRIM25 mediates its association with RIG-I [39]. Because many TRIM family proteins exert their functions by catalyzing ubiquitination, we propose that the PRYSPRY domain of TRIMs provides a critical protein interaction interface and facilitate the association between TRIMs and substrates, thus the RING domain of TRIMs can mediate ubiquitination of the associated substrates. Further investigations will provide insights into the functional significance of the PRYSPRY domain of TRIMs.

While our manuscript was in preparation, we noticed that a study of TRIM38 was published online [49]. Both this study and ours indicate that TRIM38 exterts its functions as an E3 ubiquitin ligase. Zhao et al. show that TRIM38 targets NAP1 to negatively regulate TLR3/4- and RIG-I- mediated production of IFN-β [49]. NAP1 is thought to bridge the interaction between TRIF and TBK1 [50]. Interestingly, our results indicate that TRIM38 targets TRIF. It is possible that TRIM38 may target multiple signaling molecules to control TLR3-mediated signaling. Overall, our findings show how TRIF is regulated by TRIM38 and provide new insight into the mechanism by which the TLR-mediated immune response is regulated over the course of viral infection.

## Supporting Information

Figure S1 **Expression of the transfected TRIM38.** 293T/TLR3 cells were transfected with an IFN-β-luc plasmid and TRIM38 plasmid (0, 50, 200, and 500 ng). Twenty-four hours after transfection, cells were incubated with 100 µg/ml of poly(I:C) for 4 h. Cell lysates were analyzed by immunoblot with indicated antibodies.
(TIF)

Figure S2 **Expression of TRIF, TBK1 and IKKi.** 293T cells were transfected with an IFN-β-luc plasmid, together with a plasmid expressing TRIF (A), TBK1 (B), or IKKi (C), and a TRIM38 plasmid (0, 50, 100, and 200 ng), respectively. Twenty-four hours after transfection, cell lysates were analyzed by immunoblot with indicated antibodies.
(TIF)

Figure S3 **Expression of TRIF protein in HeLa cells treated with poly(I:C).** HeLa cells were treated with 400 µg/ml poly(I:C). At indicated time points, cells were harvested and analyzed by immunoblot using the goat anti-TRIF and rabbit anti-TRIM38 antibodies. β-actin was used as an internal control.
(TIF)
