# Supplementary figures and images for: TRIM38 Negatively Regulates TLR3-Mediated IFN-β Signaling by Targeting TRIF for Degradation
Source: PLoS One. 2012 Oct 8;7(10):e46825. doi: 10.1371/journal.pone.0046825 (PMC3466175; doi:10.1371/journal.pone.0046825)

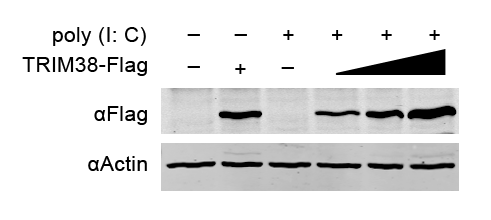

Supplement: Figure S1 — Expression of the transfected TRIM38. 293T/TLR3 cells were transfected with an IFN-β-luc plasmid and TRIM38 plasmid (0, 50, 200, and 500 ng). Twenty-four hours after transfection, cells were incubated with 100 µg/ml of poly(I:C) for 4 h. Cell lysates were analyzed by immunoblot with indicated antibodies. (TIF) [file pone.0046825.s001.tif]

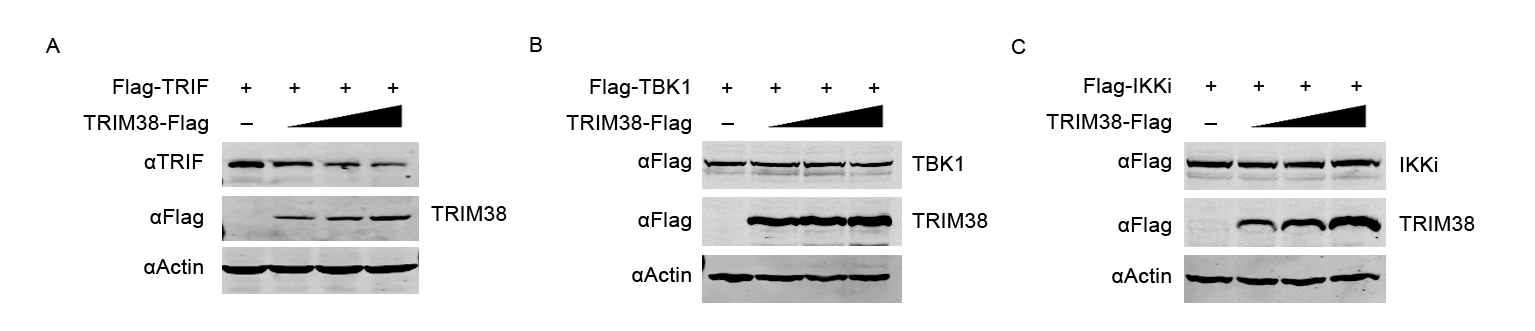

Supplement: Figure S2 — Expression of TRIF, TBK1 and IKKi. 293T cells were transfected with an IFN-β-luc plasmid, together with a plasmid expressing TRIF (A), TBK1 (B), or IKKi (C), and a TRIM38 plasmid (0, 50, 100, and 200 ng), respectively. Twenty-four hours after transfection, cell lysates were analyzed by immunoblot with indicated antibodies. (TIF) [file pone.0046825.s002.tif]

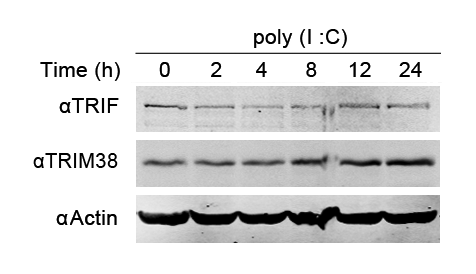

Supplement: Figure S3 — Expression of TRIF protein in HeLa cells treated with poly(I:C). HeLa cells were treated with 400 µg/ml poly(I:C). At indicated time points, cells were harvested and analyzed by immunoblot using the goat anti-TRIF and rabbit anti-TRIM38 antibodies. β-actin was used as an internal control. (TIF) [file pone.0046825.s003.tif]
